# Affective Responses of Healthcare Professionals and the General Public to Health Conditions Involving Physical Dysfunction: A Cross-Sectional Web-Based Survey of Stroke, Femoral Neck Fracture, and Spinal Cord Injury

**DOI:** 10.3390/healthcare14091202

**Published:** 2026-04-29

**Authors:** Junko Ochi, Noriyuki Kida

**Affiliations:** 1 Faculty of Health Sciences, Bukkyo University, Kyoto 604-8418, Japan; 2 Faculty of Arts and Sciences, Kyoto Institute of Technology, Kyoto 606-8585, Japan

**Keywords:** stroke, femoral neck fracture, spinal cord injury, healthcare professionals, general public, negative affect, interpersonal avoidance, stigma

## Abstract

Background/Objectives: Attitudes toward health conditions involving physical dysfunction may differ between healthcare professionals (HCPs) and the general public. This study compared affective responses to stroke, femoral neck fracture (FNF), and spinal cord injury (SCI) across both groups within a unified framework. Methods: We conducted an online cross-sectional survey of 400 members of the general public (stratified by age and gender) and 400 HCPs representing 18 professions. Affective responses were measured using scales assessing negative affect, interpersonal avoidance, and impressions (physical strength, mental resilience, and mental strength). A 2 × 3 mixed-model ANOVA (group × condition) and correlation analyses were conducted. Results: Negative affect showed a significant group × condition interaction (*p* < 0.001, ηp^2^ = 0.030). HCPs scored lower than the general public for stroke and FNF, with no significant difference for SCI. HCPs also reported lower interpersonal avoidance across all conditions. Impression scales showed no interaction; however, HCPs rated higher physical strength and mental strength, while mental resilience showed no group difference. Condition effects were significant, with a consistent hierarchy of SCI > stroke > FNF for both negative affect and interpersonal avoidance. Negative affect and interpersonal avoidance were moderately correlated in both groups. Among HCPs, FNF-related interpersonal avoidance negatively correlated with years of clinical experience. Conclusions: HCPs generally report less negative affect and interpersonal avoidance and hold more positive perceptions of patient capability than the general public. However, both groups share a negativity hierarchy across conditions, suggesting persistent perceptions associated with specific health labels.

## 1. Introduction

Diseases and disabilities are not rare occurrences affecting only a few individuals; they represent a persistent and substantial proportion of society. Japan is a “super-aged society” with an aging rate exceeding 21%, and the population aged 65 and over accounts for 29.1% of the total population [1]. Furthermore, according to the 2024 White Paper on Persons with Disabilities [2], there are an estimated 4.36 million people with physical disabilities, 1.094 million with intellectual disabilities, and 6.148 million with mental disorders, indicating that approximately 9.2% of the Japanese population lives with some form of disability. These figures demonstrate that diseases and disabilities are everyday realities within society. Under these social conditions, the perceptions and attitudes of others toward individuals with diseases and disabilities can significantly influence their social participation and the support they receive.

The social evaluation by others (negative attitudes and social distance) toward individuals with health conditions involving physical dysfunction may be related to the degree of acceptance for helping behaviors and social participation, as well as the rehabilitation process and its effectiveness. However, quantitative studies comparing different physical illnesses across multiple groups within a unified framework remain limited.

Furthermore, while a certain number of comparative studies have examined “prejudice against illness” between healthcare professionals (HCPs) and the general public, their findings are inconsistent. HCPs are not always more positive (i.e., less prejudiced) than the general public; some reports indicate little to no difference between the groups, while others even show reversed results. This variation in findings is likely attributable to differences in the occupational composition of the participants [3,4], as well as variations in the countries where the surveys were conducted, healthcare systems, cultures, and assessment scales. Moreover, most existing comparative studies have focused on mental illness or severe diseases [5,6,7,8], and research comparing physical illnesses or physical disabilities within a unified framework is extremely limited [9,10].

In addition, evidence from rehabilitation-related and primary care fields suggests that healthcare professionals’ beliefs about health conditions may influence not only attitudes but also clinical judgment and management perspectives. Studies of low back pain among physiotherapists and primary care physicians have shown that practitioners’ beliefs and treatment orientations are associated with views on diagnosis and management, as well as with clinical decisions and advice [11,12,13]. Such findings suggest that underlying cognitive and affective representations of health conditions may shape healthcare practice, highlighting the importance of examining these responses among healthcare professionals. This gap is also theoretically important. Stigma theory conceptualizes labeling, stereotyping, prejudice, and social distance as socially structured processes [14,15,16].

In addition, responses to health conditions are shaped not only by pathology itself but also by the psychological and social meanings attached to the condition [17]. Illness perception frameworks further suggest that cognitive and emotional representations of a condition influence its interpretation and the responses it evokes [18]. Taken together, these perspectives suggest that responses to condition labels may reflect not only medical features but also socially shared beliefs concerning visibility, permanence, recoverability, and causation.

Therefore, as a first report [19], the authors conducted a web-based survey targeting the general public. The conditions were selected from those with high inpatient treatment rates in Japan according to disease classification [20]. Specifically, from the categories of “Mental and behavioral disorders,” “Diseases of the circulatory system,” and “Injury, poisoning and certain other consequences of external causes,” we selected (1) stroke (cerebrovascular disease) from the “Diseases of the circulatory system” category, and (2) fracture and (3) spinal cord injury from the “Injury, poisoning and certain other consequences of external causes” category. The three conditions were selected not only because of their epidemiological relevance in Japan [20], but also because they differ in characteristics that may shape affective responses, such as visibility, perceived recoverability, causal attribution [15,16,17]. Stroke, femoral neck fracture, and spinal cord injury were therefore considered a meaningful comparative set within a unified framework. Our first report further suggested that response patterns differ by condition and that impressions are influenced not only by biological characteristics such as severity or visibility but also by the meanings and cognitive frameworks associated with each condition [19].

In this study, medical diagnostic names such as stroke, femoral neck fracture (FNF), and spinal cord injury (SCI) are collectively referred to as “health conditions” for convenience. These concepts encompass health conditions and traumas with different pathogenic mechanisms and pathologies and are not necessarily treated homogeneously as a single “disease.” The focus of this study is not on the pathology itself, but on the affective responses that arise when these condition labels are presented.

Based on the first report, the objective of this study is to compare the affective responses to these health conditions (stroke, FNF, and SCI) between HCPs and the general public within a unified framework. For the general public, the dataset from the first report was used for secondary analysis. Combined with a newly collected sample of HCPs, the study aimed to (1) verify the factor structure of the scales used in the first report among HCPs, (2) explore the presence of main effects (group differences and condition differences) and interactions for the negative affect and impression scales using a group (HCP/General) × condition (3 levels) design, and (3) examine the correlations between each scale.

## 2. Methods

### 2.1. Study Design and Procedures

This study employed a cross-sectional design using independent samples from two groups surveyed at different time points: the general public group in February 2025 and the HCPs group in August 2025. Data were collected using quota sampling from registered panel members of Rakuten Insight, Inc. (Tokyo, Japan). For each group, a target sample size of 400 was established, with pre-set quotas for eight cells consisting of four age groups (20–29, 30–39, 40–49, and 50–59 years) and gender (male and female), resulting in 50 participants per cell. Recruitment for each group was concluded once all cells reached their target numbers, yielding a final sample of 400 HCPs and 400 members of the general public (Total *N* = 800).

In the screening survey, respondents provided self-reported data regarding their nationality, age (20–59 years), gender, and professional qualifications (for the HCP group, possession of any of the 18 specified national medical licenses). Only those meeting the eligibility criteria were allowed to proceed to the main survey. These attributes were based on the survey company’s registration records and screening responses; no third-party verification, such as the submission of professional certificates, was performed (a recognized limitation of online surveys).

Prior to the commencement of the main survey, an online explanatory document was presented to all potential participants, outlining the voluntary nature of participation, the right to withdraw at any time, and the protocols for handling personal information. Only individuals who selected “I agree” were permitted to proceed (electronic informed consent). If “I do not agree” was selected, the survey was terminated, and no data were recorded. The data for the general public group were utilized in accordance with the ethical review procedures approved for the previous study [19] (Approval No. 2024-68). The survey for the HCP group was conducted following separate approval from the Ethics Committee of the Kyoto Institute of Technology (Approval No. 2025-49).

**Figure 1:**
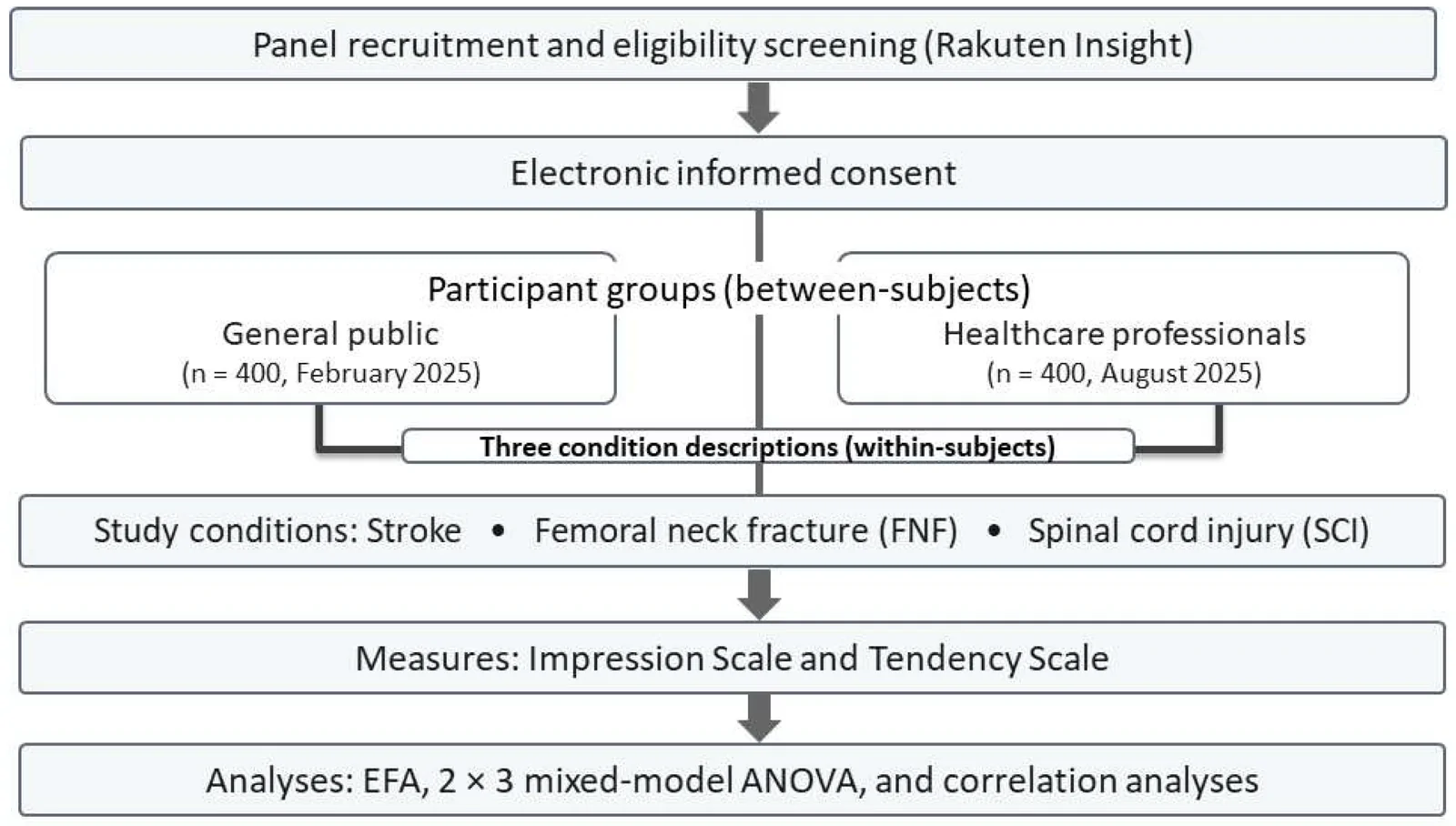
Study Design and Analytical Procedure.

Figure 1 provides a schematic overview of the study design and procedures.

### 2.2. Participants

#### 2.2.1. General Public Group (*n* = 400)

We utilized data from our previous study [19], collected in February 2025. Participants were Japanese residents aged 20–59. While the previous study restricted analysis to respondents with no personal or caregiving experience with the three health conditions (*n* = 351), the current study included all collected respondents (*n* = 400).

#### 2.2.2. HCP Group (*n* = 400)

The sample consisted of 18 professional categories (physicians, dentists, public health nurses, midwives, nurses, assistant nurses, physical therapists, occupational therapists, speech-language-hearing therapists, orthoptists, radiological technologists, clinical laboratory technicians, pharmacists, emergency medical technicians, judo therapists, acupuncturists, moxibustion therapists, and massage therapists). HCPs were defined as individuals holding national professional licenses in Japan involved in clinical activities (diagnosis, treatment, nursing, rehabilitation, or emergency response). These 18 professions shared a foundation in clinically oriented medical education. Although clinical contact frequency with the three conditions varied by profession, we analyzed the HCPs as a single group, as the study aimed to compare HCPs with the general public rather than examine differences between professions [Collection period: August 2025].

### 2.3. Study Procedures

Prior to responding, participants read explanatory texts (approximately 300 characters each) for the three health conditions: stroke, FNF, and SCI. The texts included an overview, causes, and characteristic symptoms and sequelae of each condition. Participants were instructed: “After reading the explanatory text, please select the image that best matches your impression of a person with the named condition. Assume that the person is living at home or in society while experiencing some difficulties in physical function or activities of daily living.” This instruction aimed to standardize the assumed level of functional limitation and social participation across the three conditions, thereby reducing variation in responses caused by imagining either extreme severity or complete recovery.

The three conditions were selected based on the highest inpatient utilization rates in Japan [20]. Specifically, we selected (1) stroke (cerebrovascular disease) from the “Diseases of the circulatory system” category, and (2) fracture and (3) spinal cord injury from the “Injury, poisoning, and certain other consequences of external causes” category. See Supplementary Materials for full descriptions of each condition.

While this procedure helped standardize the assumed level of functional limitation and social participation across conditions, the explanatory texts may also have influenced participants’ perceptions of the conditions.

### 2.4. Measures

Based on preliminary studies [21,22,23,24] used in our first report [19], we utilized two scales in this study. The factor structures were re-examined and scored as follows:

#### 2.4.1. Impression Scale

To assess impressions of individuals with these conditions, we used a 10-item Semantic Differential (SD) scale (Table 1). Each item consisted of bipolar adjective pairs rated on a 7-point scale (1 = very [left adjective], 2 = quite [left adjective], 3 = somewhat [left adjective], 4 = neither adjective applies, 5 = somewhat [right adjective], 6 = quite [right adjective], and 7 = very [right adjective]). Higher scores indicated more positive impressions.

Table 1 presents the original items used at the scale development stage.

#### 2.4.2. Tendency Scale

To measure attitudes and affective responses toward individuals with these conditions, we used a 10-item Likert scale (Table 2). Each item was rated on a 5-point scale (1 = not at all applicable, 2 = hardly applicable, 3 = somewhat applicable, 4 = applicable, and 5 = very applicable). Higher scores indicated stronger negative responses.

Table 2 presents the original item pool used at the scale development stage.

### 2.5. Terminology

We define prejudice as “an individual’s attitudinal response, such as negative affect or avoidance, toward a health label” [25]. Stigma is defined as “a social process where labeling, stereotyping, prejudice, and discrimination co-occur under power situations” [14]. Thus, the indicators used in this study (interpersonal avoidance, negative affect, and impressions) are categorized as aspects of prejudice (affect, behavior, and stereotypes) that constitute stigma. Note that stigma itself is not solely defined by individual attitudes but also includes social and systemic dimensions.

### 2.6. Statistical Analysis

#### 2.6.1. Factor Analysis Procedures

To examine whether the factor structure identified in our first study was replicated in this independent dataset, we performed exploratory factor analysis (EFA) on the combined sample (*N* = 800). Although EFA was used, the number of factors was specified a priori on the basis of the prior study and the theoretical framework; therefore, the analysis was not fully exploratory.

Impression Scale: We conducted EFA (maximum likelihood estimation, Promax rotation) by specifying three factors based on the theoretical framework, identifying three factors: “physical strength,” “mental resilience,” and “mental strength.”

Tendency Scale: We excluded item q10 (“I believe one can live positively even with illness or disability”) because it formed a single factor and produced a Heywood case (communalities > 1). We performed EFA (maximum likelihood estimation, Promax rotation) on the remaining nine items, identifying two factors: “negative affect” and “interpersonal avoidance.” In the Tendency scale, we reverse-coded item q2 before calculating factor scores. Scores for each factor were calculated as the item mean and used for subsequent analyses.

#### 2.6.2. Comparison of Health Conditions

We conducted 2 × 3 mixed-model ANOVAs (group [HCP/general public] × condition [stroke/FNF/SCI]) for each of the five factors. We assessed homogeneity of variance using Levene’s test and the sphericity of within-subject factors using Mauchly’s test. If the assumption of sphericity was violated, we applied the Greenhouse–Geisser (GG) correction and reported the adjusted degrees of freedom. When interaction effects were significant, we examined simple main effects and conducted post hoc comparisons with Bonferroni correction. We reported effect sizes as partial eta-squared (ηp^2^), descriptive statistics as mean ± standard deviation, and included estimated marginal means (EMM) with 95% CI where appropriate.

Because the factor scores were calculated as mean scores from multiple items and the sample sizes were large in both groups, parametric analyses were considered appropriate.

#### 2.6.3. Correlations Between Scales

We analyzed the correlations between scale scores using Pearson’s correlation coefficient. We examined the correlations for both the HCP and general public groups to compare the characteristics of associations between factors. Furthermore, for the HCP group, we performed correlation analysis to examine associations between scale scores, years of clinical experience, and age. Statistical analyses were performed using IBM SPSS Statistics version 30.0 (IBM Corp., Armonk, NY, USA).

## 3. Results

### 3.1. Results of Factor Analysis

#### 3.1.1. Impression Scale for Illness/Patients

Based on the results of our previous study, we specified three factors and confirmed that the theoretically interpretable factor structure was replicated. The three factors were identified as “Physical Strength,” “Mental Resilience,” and “Mental Strength,” representing the three dimensions of perceived physical strength, mental resilience, and mental strength, respectively (Table 3).

#### 3.1.2. Tendency Scale for Illness/Patients

Based on the factor loadings, two factors were identified: negative affect (e.g., anxiety and sadness) and interpersonal avoidance (e.g., hesitation to interact with or help the person) (Table 4).

Raw item scores were used for the factor analysis. Prior to computing factor scores, the reverse-worded item (q2) in the Tendency scale was reverse-coded.

### 3.2. Results of Group Comparisons

#### 3.2.1. Impression Scale (Figure 2, Table 5)

For all three factors, the interaction between group (HCP/general public) and the three conditions (stroke/FNF/SCI) was not significant [Physical Strength: GG F(1.886, 1504.758) = 0.175, *p* = 0.827, ηp^2^ = 0.000; Mental Resilience: GG F(1.761, 1405.313) = 3.128, *p* = 0.051, ηp^2^ = 0.004; Mental Strength: GG F(1.924, 1534.981) = 0.363, *p* = 0.687, ηp^2^ = 0.000]. Therefore, we interpreted the main effects.

**Table 5:** Comparison of Impression Scale scores by group and condition.

		Group (Mean ± SD)	
Factor	Disease	HCP	General Public
physical strength	SCI	3.778 ± 0.969	3.643 ± 0.967
	stroke	3.939 ± 0.853	3.809 ± 0.984
	FNF	3.929 ± 0.823	3.766 ± 0.891
mental resilience	SCI	3.778 ± 0.922	3.844 ± 0.768
	stroke	3.708 ± 1.047	3.714 ± 0.913
	FNF	3.978 ± 0.767	3.896 ± 0.753
mental strength	SCI	3.908 ± 0.884	3.795 ± 0.911
	stroke	4.074 ± 0.842	3.936 ± 0.910
	FNF	4.019 ± 0.731	3.855 ± 0.834

Abbreviations: HCP, healthcare professional; SCI, spinal cord injury; FNF, femoral neck fracture.

**Figure 2:**
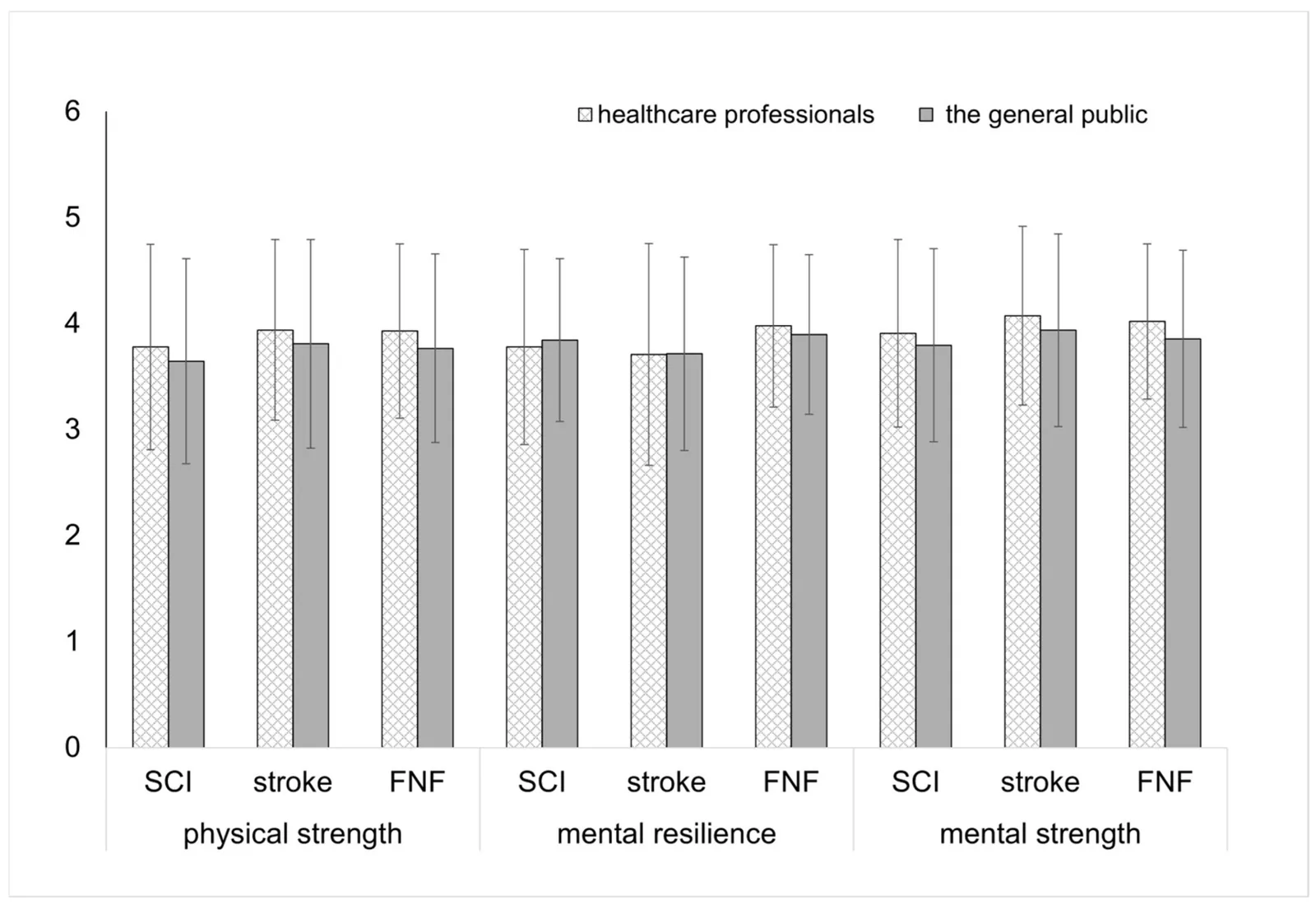
Mean scores of the Impression Scale across three conditions. Abbreviations: SCI, spinal cord injury; FNF, femoral neck fracture.

(1) Main effect of group

HCPs scored significantly higher than the general public on Physical Strength and Mental Strength [Physical Strength: F(1, 798) = 6.774, *p* = 0.009, ηp^2^ = 0.008; Mental Strength: F(1, 798) = 7.894, *p* = 0.005, ηp^2^ = 0.010]. No group difference was observed for Mental Resilience [F(1, 798) = 0.004, *p* = 0.948, ηp^2^ = 0.000].

(2) Main effect of the three conditions (GG correction)

Significant for all factors. Physical Strength: F(1.886, 1504.758) = 17.404, *p* < 0.001; SCI < stroke = FNF. Mental Resilience: F(1.761, 1405.313) = 29.014, *p* < 0.001; FNF > SCI > stroke. Mental Strength: F(1.924, 1534.981) = 12.933, *p* < 0.001; SCI < (stroke = FNF). Descriptive statistics (mean ± SD) are shown in Table 5.

#### 3.2.2. Tendency Scale (Figure 3, Table 6)

Note: Scores range from 1 to 5, with higher scores indicating stronger negative responses.

**Table 6:** Comparison of Tendency Scale scores by group and condition.

		Group (Mean ± SD)	
Factor	Disease	HCP	General Public
interpersonal avoidance	SCI	2.632 ± 0.761	2.921 ± 0.716
	stroke	2.450 ± 0.680	2.803 ± 0.676
	FNF	2.339 ± 0.734	2.769 ± 0.731
negative affect	SCI	3.151 ± 0.948	3.274 ± 0.922
	stroke	2.890 ± 0.772	3.157 ± 0.827
	FNF	2.690 ± 0.833	3.126 ± 0.884

Abbreviations: HCP, healthcare professional; SCI, spinal cord injury; FNF, femoral neck fracture.

**Figure 3:**
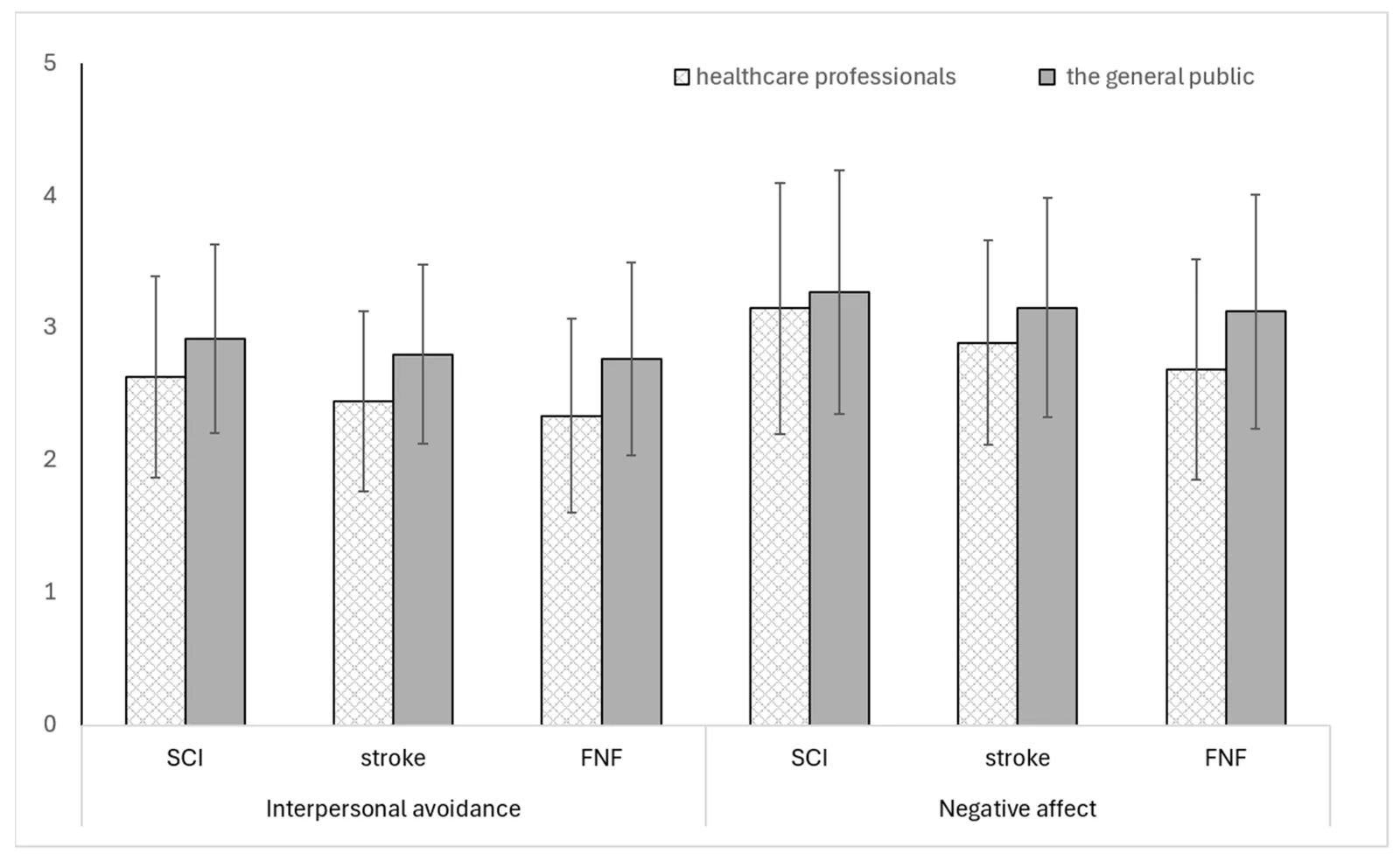
Mean scores of the Tendency Scale across three conditions.

(1) Negative affect

The group × condition interaction was significant (GG correction: F(1.906, 1521.087) = 24.538, *p* < 0.001, ηp^2^ = 0.030). Simple main effect analysis revealed that for stroke and FNF, HCPs scored lower than the general public (both *p* < 0.001), while no group difference was observed for SCI (*p* = 0.064). Within-group differences for conditions were: HCPs, SCI > stroke > FNF (all *p* < 0.001); general public, SCI > (stroke = FNF) [SCI vs. stroke, SCI vs. FNF: *p* ≤ 0.001; stroke vs. FNF: n.s.]. The main effect of the group was significant, with HCPs scoring lower than the general public overall (F(1, 798) = 24.540, *p* < 0.001, ηp^2^ = 0.030; EMM [HCP = 2.911, general public = 3.186]). The main effect of the diagnostic label was also significant (F(1.906, 1521.087) = 94.320, *p* < 0.001, ηp^2^ = 0.106; EMM [3.213 > 3.024 > 2.908]), with SCI being perceived as the most negative.

(2) Interpersonal avoidance

The group × condition interaction was significant (Sphericity assumed: Mauchly *p* = 0.091; F(2, 1596) = 6.318, *p* = 0.002, ηp^2^ = 0.008). Simple main effect analysis showed that HCPs scored lower than the general public for all three conditions (all *p* < 0.001). Within-group condition differences were: HCPs, SCI > stroke > FNF (all *p* < 0.001); general public, SCI > (stroke = FNF) [SCI vs. stroke, SCI vs. FNF: *p* < 0.001; stroke vs. FNF: n.s.]. The main effect of the group was significant, with HCPs scoring lower than the general public (F(1, 798) = 62.634, *p* < 0.001, ηp^2^ = 0.073; EMM [HCP = 2.473, general public = 2.831]). The main effect of the condition was also significant (GG correction: F(1.988, 1586.480) = 64.667, *p* < 0.001, ηp^2^ = 0.075), with SCI > stroke > FNF (EMM: SCI = 2.776, stroke = 2.626, FNF = 2.554).

### 3.3. Correlation Analysis (Table 7 and Table 8)

Note: Since the Impression and Tendency scales have opposite scoring directions, a negative correlation indicates that higher positive impressions correlate with lower prejudice tendencies.

**Table 7:** Correlations among factors in the general public group.

		Interpersonal Avoidance	Negative Affect	Physical Strength	Mental Resilience	Mental Strength
stroke	interpersonal avoidance	1				
	negative affect	0.474 **	1			
	physical strength	−0.224 **	−0.227 **	1		
	mental resilience	−0.207 **	−0.286 **	0.291 **	1	
	mental strength	−0.204 **	−0.122 *	0.658 **	0.342 **	1
FNF	interpersonal avoidance	1				
	negative affect	0.504 **	1			
	physical strength	−0.065	−0.262 **	1		
	mental resilience	−0.120 *	−0.199 **	0.424 **	1	
	mental strength	−0.121 *	−0.194 **	0.730 **	0.479 **	1
SCI	interpersonal avoidance	1				
	negative affect	0.564 **	1			
	physical strength	−0.252 **	−0.398 **	1		
	mental resilience	−0.220 **	−0.205 **	0.463 **	1	
	mental strength	−0.255 **	−0.302 **	0.779 **	0.603 **	1

** *p* < 0.01, * *p* < 0.05.

**Table 8:** Correlations among factors in the healthcare professional group.

		Interpersonal Avoidance	Negative Affect	Physical Strength	Mental Resilience	Mental Strength
stroke	interpersonal avoidance	1				
	negative affect	0.448 **	1			
	physical strength	−0.108 *	−0.138 **	1		
	mental resilience	−0.105 *	−0.153 **	0.356 **	1	
	mental strength	−0.139 **	0.013	0.575 **	0.413 **	1
FNF	interpersonal avoidance	1				
	negative affect	0.536 **	1			
	physical strength	−0.033	−0.133 **	1		
	mental resilience	−0.161 **	−0.131 **	0.442 **	1	
	mental strength	−0.130 **	−0.080	0.732 **	0.631 **	1
SCI	interpersonal avoidance	1				
	negative affect	0.495 **	1			
	physical strength	−0.195 **	−0.337 **	1		
	mental resilience	−0.168 **	−0.218 **	0.420 **	1	
	mental strength	−0.200 **	−0.229 **	0.760 **	0.573 **	1

** *p* < 0.01, * *p* < 0.05.

#### 3.3.1. Negative Affect Tendency and Interpersonal Avoidance Tendency

Significant positive correlations were observed for all three conditions in both groups (General public: r = 0.474–0.564, *p* < 0.01; HCPs: r = 0.448–0.536, *p* < 0.01).

#### 3.3.2. Negative Affect Tendency and Impression Scale

Negative correlations were found for all conditions in the general public group (r = −0.122 to −0.398). However, in the HCP group, no correlation was observed with Mental Strength for some conditions (stroke: r = 0.013; FNF: r = −0.08).

#### 3.3.3. Interpersonal Avoidance Tendency and Impression Scale

No correlation was observed for Physical Strength for FNF in either the HCP (r = −0.033) or the general public (r = −0.065) group. Significant correlations were observed for the other conditions.

#### 3.3.4. Clinical Experience (HCPs)

Partial correlation analysis controlling for age revealed a negative correlation between years of clinical experience and interpersonal avoidance for FNF (r = −0.131, *p* = 0.009). No other significant associations were found between clinical experience and the other subscales.

## 4. Discussion

This study compared the affective responses of HCPs and the general public to the names of health conditions (stroke, FNF, and SCI) within a unified framework.

### 4.1. Interpretation of Group Differences

The scores for the prejudice indices, “negative affect” and “interpersonal avoidance,” were on average lower in the HCP group than in the general public group. However, for negative affect, a significant group × condition interaction was observed; HCPs scored lower for stroke and FNF, whereas no significant group difference was found for SCI. Regarding “interpersonal avoidance,” HCPs scored significantly lower across all three conditions. These results suggest that professional training may promote a clearer understanding of etiology, prognosis, and available support resources. Furthermore, the clarification of professional roles may alleviate negative affect, which in turn may suppress feelings of interpersonal avoidance. This finding is consistent with previous research [9] showing that nurses and nursing students exhibit more positive attitudes toward people with disabilities compared to the general public or other students. Additionally, the professional sense of norms (e.g., fairness and care) among HCPs is thought to reinforce this positive orientation.

Regarding the impression scales, HCPs perceived “physical strength” and “mental strength” more positively, while no group difference was observed for “mental resilience.” This distinction may arise because the factors “physical strength” and “mental strength” reflect perceptions of personal competence, such as physical vitality, willpower, and persistence. In contrast, “mental resilience” reflects an impression dimension closer to personality traits, such as flexibility, adaptability, and composure. It is possible that both groups reached a similar evaluation for “mental resilience” because the condition labels alone provided insufficient cues to estimate these specific personality traits. These findings suggest that for HCPs, specialized education, knowledge, and contact with patients may be associated with reduced negative affect, shortened social distance, and a more positive perception of the individuals’ perceived competence. These interpretations should be considered with caution, as factors such as professional training and clinical experience were not directly measured in the present study. In addition, although several group differences were statistically significant, the observed effect sizes were relatively small.

### 4.2. Correlations Among Factors

#### 4.2.1. Interpersonal Avoidance and Negative Affect

A moderate positive correlation was observed between “interpersonal avoidance” and “negative affect” across both groups and all three health conditions. This result is consistent with the classical tripartite model of attitudes [26], which posits that the three components of attitude—cognitive, affective, and behavioral—are interrelated. Specifically, it indicates that as affective responses such as aversion or anxiety toward a target intensify, the corresponding behavioral tendency to avoid or maintain distance also increases.

#### 4.2.2. Negative Affect and the Three Impression Factors (Mental Strength, Physical Strength, and Mental Resilience)

Among the general public, results were consistent: more positive impressions were associated with lower levels of prejudice across all factors. However, in the HCP group, no correlation was found between “negative affect” and “mental strength” for stroke and FNF. This suggests that for HCPs, affective responses may be regulated by factors other than the impression of “mental strength,” such as specialized professional knowledge or a sense of professional norms.

#### 4.2.3. Interpersonal Avoidance and the Three Impression Factors

In both groups, no correlation was observed between “interpersonal avoidance” and “physical strength” exclusively for FNF. This suggests that the low level of prejudice toward FNF may be determined by preexisting schemas (illness representations) associated with the condition label rather than being formed through immediate impressions. In Japan, the high prevalence of FNF [20] likely provides frequent opportunities for contact or information. Furthermore, because the clinical presentation, standardized management (surgical intervention and early rehabilitation), and prognosis of FNF are widely recognized, it is easily perceived as “recoverable.” A study in the United Kingdom reported that the general public often underestimates the severity of hip fractures (inpatient duration, mortality, and functional decline) [27]; regardless of the accuracy of such perceptions, FNF tends to be viewed relatively positively or optimistically. Taken together, the lack of association between “interpersonal avoidance” and perceived competence for FNF observed in this study suggests that social distance may be stabilized at a low level by existing illness schemas—characterized by high frequency, standardized treatment, and perceived recoverability—independent of impressions regarding the individual’s competence.

Additionally, for the correlation between years of clinical experience and each factor in the HCP group, a weak negative correlation was found between “clinical experience” and “interpersonal avoidance” only for FNF. Increased contact with patients may reduce social distance. However, as background factors such as specific profession, contact frequency, and quality were not fully controlled, this interpretation remains preliminary and warrants further investigation.

### 4.3. Differences Among Health Conditions (Hierarchy Based on Condition Characteristics)

This study examined three physical health conditions with varying visibility, etiology, and primary affected age groups. For “negative affect,” both groups followed the hierarchy of SCI > stroke > FNF. However, while these three conditions were distinctly separated in the HCP group, stroke and FNF were evaluated at equivalent levels in the general public group. A similar pattern was observed for “interpersonal avoidance,” with the hierarchy of SCI > stroke > FNF, though again, no significant difference was found between stroke and FNF in the general public.

The results for the impression scales were generally consistent with this pattern: SCI was evaluated most negatively for “physical strength” and “mental strength,” while FNF was perceived most positively for “mental resilience” (the order of SCI and stroke for mental resilience varied by group). This consistent pattern aligns with stigma theories suggesting that attitude components are determined by condition characteristics such as visibility, persistence, recoverability, and causal attribution [15,16]. Specifically, SCI is characterized by high visibility of the disability and assistive devices (e.g., wheelchairs) and high permanence, which tends to facilitate “segregation”—a tendency to draw boundaries and distance oneself from the group—and increases social distance. Furthermore, while the primary cause of traumatic SCI in Japan has shifted from traffic accidents to falls on level ground in recent years [28], the general public’s recall is still dominated by the image of “traffic accidents involving young people,” which may be associated with perceived risk-taking behaviors. Although this study focuses on the attitudes of others, the fact that SCI-specific stigma scales have been developed and disseminated based on patient reports [29,30] underscores that stigma experiences manifest as clinical and social disadvantages. In light of these condition characteristics and the strength of the associated social images, SCI may consistently elicit stronger negative responses in both groups, and group differences in negative affect may be less likely to emerge. In other words, affective responses toward SCI may be relatively robust and less susceptible to modification through professional education or clinical experience. This suggests that reactions to SCI are shaped not only by individual differences in knowledge but also by factors such as the visibility of disability, the perceived permanence of functional impairment, causal attributions, and socially shared illness representations. Therefore, understanding and mitigating negative responses toward SCI requires consideration not only of knowledge-based factors but also of how such social images are formed and maintained.

Regarding stroke, while it is one of the circulatory diseases with a high incidence rate in Japan [20] and information and awareness regarding its onset and sequelae have accumulated, biases and deficiencies in knowledge concerning symptom recognition, acute response, and recovery prognosis persist [31,32]. This suggests that evaluations of the condition remain unstable. As for FNF, as described in Section 4.1, the high prevalence, relatively frequent opportunities for contact, and the widely shared understanding of standardized treatment and recovery prospects likely contributed to its relatively positive evaluation among the three conditions.

### 4.4. Discrimination Between Health Conditions Among Healthcare Professionals

As indicated by the Group × Condition interaction, “negative affect” and “interpersonal avoidance” showed the most negative values for SCI in both groups. However, while no significant difference was observed between stroke and FNF in the general public group, a clear, stepwise hierarchy of SCI > stroke > FNF was found within the HCP group. This result suggests a duality: while professional knowledge and increased clinical experience suppress overall prejudice (i.e., negative affect and social distance), the refinement of specific knowledge regarding the risks and support systems associated with each condition leads to more distinct discrimination between different condition labels. This interpretation is consistent with the framework that views stigma as a social process [14], as well as findings indicating that cognitive evaluations—such as controllability and attribution of responsibility—influence attitude formation [16]. Furthermore, the observed differences in evaluations across conditions among HCPs align with recent reviews suggesting that implicit biases (unconscious attitudes and preconceptions) can influence clinical judgment and patient experiences [33].

In summary, HCPs consistently exhibited more positive responses than the general public to negative affect, interpersonal avoidance, and perceived competence (physical and mental strength). However, the hierarchy of negative reactions (SCI > stroke > FNF) and differences in impressions were generally shared by both groups, indicating that discrimination based on condition characteristics remains. Correlation analysis revealed that, in addition to the link between affect and avoidance, there was a unique lack of association between “physical strength” and interpersonal avoidance exclusively for FNF. These findings suggest that illness schemas and contact experiences—as evidenced by the weak negative correlation between years of clinical experience and interpersonal avoidance in HCPs—play a significant role in shaping attitudes toward these health conditions.

### 4.5. Limitations

This study has several limitations. It was a cross-sectional study based on self-reported subjective reactions and therefore did not permit causal interpretation or capture the broader social and structural dimensions of stigma. In addition, both groups were recruited through an online panel and may not be fully representative of the broader population, and the professional status of HCP participants was based on self-reported screening information without external verification. Because the general public and HCP groups were surveyed at different time points, time-related bias in the between-group comparison cannot be excluded. Finally, although several group differences were statistically significant, the effect sizes were generally small, and profession-specific differences within the HCP group may have been obscured.

## 5. Conclusions

This study provides preliminary evidence by comparing responses of HCPs and the general public to three specific health conditions (stroke, FNF, and SCI) using a unified design. The observed hierarchy of evaluations is consistent with condition characteristics—such as visibility, persistence, recoverability, and causal attribution—as well as social illness schemas, suggesting that attitudes are shaped by these cognitive dimensions. Overall, HCPs showed less negative affect and interpersonal avoidance than the general public, but both groups shared a similar hierarchy of responses across conditions, especially the relatively negative evaluation of SCI. Future research should examine the quality of contact and differences in professional expertise across occupations to clarify the mechanisms through which condition characteristics influence attitude formation.

## Figures and Tables

**Table 1:** Items of the Impression Scale.

No.	Left End (1)	Right End (7)
Q1	emotional	calm
Q2	self-centered	cooperative
Q3	stubborn	flexible
Q4	easily gives up	persevering
Q5	timid	brave
Q6	weak-willed	strong-willed
Q7	lifeless	lively
Q8	apathetic	energetic
Q9	frail	robust
Q10	feeble	vigorous

**Table 2:** Items of the Tendency scale.

No.	Item
q1	It takes a great deal of effort to interact with a patient.
q2	I can interact naturally with a patient.
q3	It is difficult to communicate with a patient.
q4	I hesitate to lend a hand to a patient.
q5	I feel that patients live in a world different from mine.
q6	I feel that having an illness or disability is frightening.
q7	I feel a sense of helplessness about illness or disability.
q8	I feel anxiety about illness.
q9	I feel sadness about illness.
q10	I feel that one can live positively even with an illness or disability.

**Table 3:** Factor analysis of the Impression Scale.

			1	2	3
Factor 1: physical strength					
Q9	frail	robust	0.930	0.052	−0.110
Q10	feeble	vigorous	0.891	−0.032	−0.010
Q8	apathetic	energetic	0.710	0.015	0.173
Q7	lifeless	lively	0.577	−0.025	0.308
Factor 2: mental resilience					
Q3	stubborn	flexible	0.089	0.856	−0.139
Q2	self-centered	cooperative	−0.084	0.794	0.080
Q1	emotional	calm	0.004	0.726	0.082
Factor 3: mental strength					
Q5	timid	brave	0.041	−0.009	0.811
Q4	easily gives up	persevering	0.015	0.212	0.612
Q6	weak-willed	strong-willed	0.160	−0.080	0.607

**Table 4:** Factor analysis of the Tendency Scale.

		1	2
Factor 1: negative affect			
q8	I feel anxiety about illness.	0.912	−0.148
q9	I feel sadness about illness.	0.871	−0.130
q6	I feel that having an illness or disability is frightening.	0.786	0.037
q7	I feel a sense of helplessness about illness or disability.	0.734	0.153
q1	It takes a great deal of effort to interact with a patient.	0.471	0.310
Factor 2: interpersonal avoidance			
q4	I hesitate to lend a hand to a patient.	−0.129	0.852
q5	I feel that patients live in a world different from mine.	0.063	0.722
q3	It is difficult to communicate with a patient.	0.141	0.645
q2	I can interact naturally with a patient.	0.082	−0.386

## Data Availability

The datasets analyzed during the current study are not publicly available due to privacy considerations but are available from the corresponding author on reasonable request.

## Supplementary Materials

The following supporting information can be downloaded at: https://www.mdpi.com/article/10.3390/healthcare14091202/s1, File S1: Full descriptions of stroke, femoral neck fracture, and spinal cord injury used in the survey.

## Abbreviations

The following abbreviations are used in this manuscript:

EMM	Estimated marginal means
GG	Greenhouse–Geisser
FNF	Femoral neck fracture
