# Correction: Ochi, J.; Kida, N. Affective Responses of Healthcare Professionals and the General Public to Health Conditions Involving Physical Dysfunction: A Cross-Sectional Web-Based Survey of Stroke, Femoral Neck Fracture, and Spinal Cord Injury. *Healthcare* 2026, *14*, 1202

**DOI:** 10.3390/healthcare14213582

**Published:** 2026-10-23

**Authors:** Junko Ochi, Noriyuki Kida

**Affiliations:** 1 Faculty of Health Sciences, Bukkyo University, Kyoto 604-8418, Japan; 2 Faculty of Arts and Sciences, Kyoto Institute of Technology, Kyoto 606-8585, Japan

## 1. Error in Table

In the original publication [1], there was a mistake in Table 4 as published. The item labels and factor loadings for Factor 2 were misaligned. The corrected Table 4 appears below.

There was a mistake in Table 6 as published. The labels “negative affect” and “interpersonal avoidance” were incorrectly associated with the corresponding values. The corrected Table 6 appears below.

## 2. Error in Figure

In the original publication [1], there was a mistake in Figure 3. The corrected Figure 3 appears below.

The authors state that the scientific conclusions are unaffected. This correction was approved by the Academic Editor. The original publication has also been updated.

## Figures and Tables

**Figure 3:**
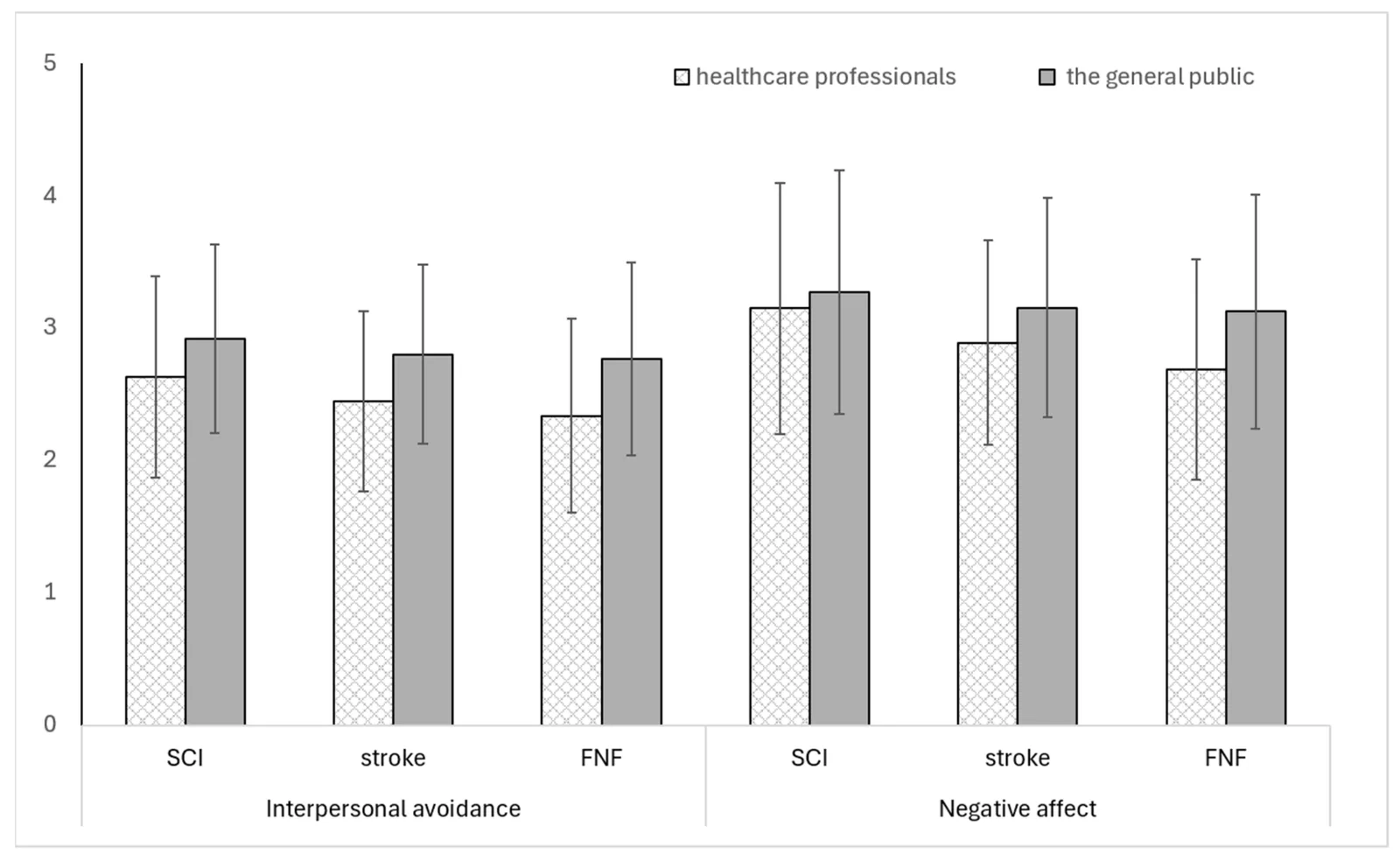
Mean scores of the Tendency Scale across three conditions.

**Table 4:** Factor analysis of the Tendency Scale.

		1	2
Factor 1: negative affect			
q8	I feel anxiety about illness.	0.912	−0.148
q9	I feel sadness about illness.	0.871	−0.130
q6	I feel that having an illness or disability is frightening.	0.786	0.037
q7	I feel a sense of helplessness about illness or disability.	0.734	0.153
q1	It takes a great deal of effort to interact with a patient.	0.471	0.310
Factor 2: interpersonal avoidance			
q4	I hesitate to lend a hand to a patient.	−0.129	0.852
q5	I feel that patients live in a world different from mine.	0.063	0.722
q3	It is difficult to communicate with a patient.	0.141	0.645
q2	I can interact naturally with a patient.	0.082	−0.386

**Table 6:** Comparison of Tendency Scale scores by group and condition.

		Group (Mean ± SD)	
Factor	Disease	HCP	General Public
interpersonal avoidance	SCI	2.632 ± 0.761	2.921 ± 0.716
	stroke	2.450 ± 0.680	2.803 ± 0.676
	FNF	2.339 ± 0.734	2.769 ± 0.731
negative affect	SCI	3.151 ± 0.948	3.274 ± 0.922
	stroke	2.890 ± 0.772	3.157 ± 0.827
	FNF	2.690 ± 0.833	3.126 ± 0.884

Abbreviations: HCP, healthcare professional; SCI, spinal cord injury; FNF, femoral neck fracture.
