# Supplementary material for: Affective Responses of Healthcare Professionals and the General Public to Health Conditions Involving Physical Dysfunction: A Cross-Sectional Web-Based Survey of Stroke, Femoral Neck Fracture, and Spinal Cord Injury
Source: Healthcare (Basel). 2026 Apr 29;14(9):1202. doi: 10.3390/healthcare14091202 (PMC13163287; doi:10.3390/healthcare14091202)
Supplement: Supplementary file 1 [file healthcare-14-01202-s001.zip › healthcare-4240366-supplementary.pdf]

Stroke

Stroke (a general term for conditions caused by problems with the blood vessels in the brain) can leave patients with residual symptoms, such as limb paralysis or speech impairments. In addition to visible impairments such as limb paralysis, there are also impairments that are not immediately apparent, such as memory loss and decreased attention span (known as higher brain dysfunction). Higher-level cognitive dysfunction refers to impairments including memory impairment (difficulty memorizing new information or recalling past memories), attention deficits (difficulty concentrating or focusing on multiple tasks), language deficits (difficulty understanding or speaking), visual perception deficits (difficulty recognizing objects or people), executive function deficits (impaired ability to plan, execute tasks, or solve problems), and social behavior deficits (problems with interpersonal relationships or social behavior).

Femoral Neck Fracture

A femoral neck fracture is a fracture of the part of the femur located at the base of the thigh. The human body is supported by the femur and the pelvis (hip joint), and the femur curves just below the hip joint. Because the curved part of the femoral neck is subjected to significant stress during falls and other incidents, fractures frequently occur in this area. Among the older adults in particular, these fractures are common due to reduced bone density and muscle strength resulting from decreased physical activity. A fracture can prevent a person from standing or walking, and even after treatment, it can lead to a bedridden state, significantly impairing quality of life and daily functioning.

Spinal Cord Injury

A spinal cord injury refers to damage to the spinal cord, which runs through the spine. Causes of injury include compression or damage resulting from traffic accidents, sports injuries, pathological causes (such as tumors or infections), and vascular abnormalities. Since the spinal cord is a vital bundle of nerves that transmits signals from the brain to various parts of the body, symptoms vary depending on the location and severity of the injury. For example, a cervical (neck) injury can cause quadriplegia (inability to move all four limbs) or respiratory distress, and may require a ventilator to sustain life. In addition, with injuries to the thoracic spine (the chest area), arm function is often preserved, but sensation and motor function in the lower body may be lost. Injuries to the lumbar spine (the lower back area) can result in paralysis of the lower body and may also affect bladder and bowel control.
